# First-line gemcitabine with cisplatin or epirubicin in advanced non-small-cell lung cancer: a phase III trial

**DOI:** 10.1038/sj.bjc.6601283

**Published:** 2003-09-30

**Authors:** F M Wachters, J W G van Putten, H Kramer, Z Erjavec, P Eppinga, J H Strijbos, G P J de Leede, H M Boezen, E G E de Vries, H J M Groen

**Affiliations:** 1 Department of Pulmonary Diseases, University Hospital Groningen, PO Box 30.001, 9700 RB Groningen, The Netherlands; 2 Department of Internal Medicine, Delfzicht Hospital, Jachtlaan 50, 9934 JD Delfzijl, The Netherlands; 3 Department of Pulmonary Diseases, Nij Smellinghe Hospital, Compagnonsplein 1, 9202 NN Drachten, The Netherlands; 4 Department of Pulmonary Diseases, Bethesda Hospital, Amshoffweg 1, 7909 AA Hoogeveen, The Netherlands; 5 Department of Epidemiology and Statistics, University of Groningen, Antonius Deusinglaan 1, 9713 AV Groningen, The Netherlands; 6 Department of Medical Oncology, University Hospital Groningen, PO Box 30.001, 9700 RB Groningen, The Netherlands

**Keywords:** non-small-cell lung cancer, cisplatin, epirubicin, gemcitabine, phase III trial, chemotherapy

## Abstract

The purpose of our study was to compare progression-free survival and quality of life (QOL) after cisplatin–gemcitabine (CG) or epirubicin–gemcitabine (EG) in chemotherapy-naive patients with unresectable non-small-cell lung cancer. Patients (*n*=240) were randomised to receive gemcitabine 1125 mg m^−2^ (days 1 and 8) plus either cisplatin 80 mg m^−2^ (day 2) or epirubicin 100 mg m^−2^ (day 1) every 3 weeks for a maximum of five cycles. Eligible patients had normal organ functions and Eastern Cooperative Oncology Group performance status ⩽2. QOL was measured with European Organisation for Research and Treatment of Cancer QLQ-C30 and LC13 questionnaires. There were no significant differences in median progression-free survival (CG 26 weeks, EG 23 weeks), median overall survival (CG 43 weeks, EG 36 weeks), or tumour response rates (CG 46%, EG 36%). Toxicity was mainly haematologic. In the EG arm granulocytopenia occurred more frequently, leading to more febrile neutropenia. Also, elevation of serum transaminases, mucositis, fever, and decline in LVEF were more common in the EG arm. In the CG arm, more patients experienced elevated serum creatinine levels, sensory neuropathy, nausea, and vomiting. Global QOL was not different in both arms. Progression-free survival, overall survival, response rate, and QOL were not different between both arms; however, overall toxicity was more severe in the EG arm.

Platinum-based chemotherapy is the standard treatment for patients with advanced non-small-cell lung cancer (NSCLC) who have a good performance status (American Society of Clinical Oncology, 1997). A meta-analysis published in 1995 showed that cisplatin-based chemotherapy induces 27% reduction in the risk of death, 10% improvement in survival at 1 year, and an absolute increase in median survival of 1.5 months compared to best supportive care (Non-small Cell Lung Cancer Collaborative Group, 1995). Since then, several new chemotherapeutic agents in combination with platinum have been investigated for their beneficial effect in patients with NSCLC. Phase II studies of new drugs in combination with platinum, published between 1992 and 1997 and reviewed by Bunn and Kelly, showed a prolonged survival from about 4 to 10 months, a prolonged 1-year survival rate from 10 to 40–50%, and an improved quality of life (QOL) (Bunn and Kelly, 1998).

In spite of the fact that cisplatin-containing regimens are currently considered the treatment of choice for advanced NSCLC, cisplatin has several disadvantages such as nephro-, neuro-, and ototoxicity. Therefore, nonplatinum-containing regimens have been studied to find less toxic therapies with similar efficacy. Phase III studies have reported similar response rates and overall survival in cisplatin and noncisplatin-based regimens. However, in the majority of these trials the nonplatinum regimens had a more favourable toxicity profile (Gatzemeier *et al*, 1991; Gridelli *et al*, 1996; Georgoulias *et al*, 2001; Kosmidis *et al*, 2002; Sculier *et al*, 2002).

Gemcitabine, a nucleoside analogue, has shown activity against NSCLC as a single agent. Phase II studies showed response rates between 18–26% and a mild toxicity profile (Anderson *et al*, 1994; Gatzemeier *et al*, 1996; Halme *et al*, 1997; Yokoyama *et al*, 1997; Perng *et al*, 1997; Takada *et al*, 1998; Zatloukal *et al*, 1998; Ten Bokkel Huinink *et al*, 1999). The combination of cisplatin and gemcitabine in NSCLC has been evaluated in phase III trials, reporting response rates of 22–41% and median survival between 8.1 and 9.7 months (Cardenal *et al*, 1999; Crino *et al*, 1999; Comella *et al*, 2000; Sandler *et al*, 2000; Schiller *et al*, 2002).

As a single-agent epirubicin, the 4′ epimer of the anthracycline antibiotic doxorubicin, showed tumour response rates from 17 up to 36% in NSCLC (Wils *et al*, 1990; Feld *et al*, 1992; Smit *et al*, 1992). The main toxicities of epirubicin are myelosuppression, mucositis, and cardiomyopathy (Wils *et al*, 1990; Feld *et al*, 1992; Smit *et al*, 1992). In our institution, the activity of epirubicin combined with gemcitabine was studied in phases I and II study (Van Putten *et al*, 2000). In this trial, a dose of 1125 mg m^−2^ gemcitabine on days 1 and 8 of each 21-day cycle was chosen since this dose leads to a dose intensity of 750 mg m^−2^ week^−1^, which is similar as the schedule in which single-agent gemcitabine 1000 mg m^−2^ is given weekly for 3 consecutive weeks in a 28-day schedule. The nadir of epirubicin is expected 12–15 days after administration; therefore, gemcitabine was omitted on day 15. The phase II trial continued with a dose of 100 mg m^−2^ epirubicin because in the preceeding phase I dose-escalation trial, a maximum tolerated dose of 120 mg m^−2^ was reached. The haematologic toxicity of this regimen was acceptable with granulocytopenia grade 4 in 33% and thrombocytopenia grade 4 in 12% of the cycles. Febrile neutropenia occurred in 14% of patients. Nonhaematologic toxicity was mainly mucositis grade 2 and 3 in 35% of patients. Cardiotoxicity measured as a significant decrease of left-ventricular ejection fraction (LVEF) was observed in 7% of patients. The tumour response rate was 49% and the median survival time was 42 weeks (Van Putten *et al*, 2000).

Manageable toxicity and high response rates of the epirubicin–gemcitabine regimen were the background for initiating this phase III trial. The aim of this phase III trial was to compare the efficacy and safety of gemcitabine in combination with either cisplatin (standard arm) or epirubicin. Epirubicin in combination with gemcitabine (EG) was administered as an outpatient regimen, the cisplatin–gemcitabine (CG) combination was given as a short inpatient regimen as is often done in European countries. The American Society of Clinical Oncology guidelines (published in 1997) recommended two to eight cycles of platinum-based treatment in advanced NSCLC. We chose a maximum of five cycles. Progression-free survival was the primary end point of the study. Overall survival, response rate, toxicity, and QOL were secondary end points.

## PATIENTS AND METHODS

### Patient selection

Patients were included if they had histological or cytological diagnosis of unresectable stage III or stage IV NSCLC and at least one measurable or evaluable tumour lesion on physical examination, chest X-ray, or chest CT. No prior chemotherapy was allowed and radiotherapy was permitted as long as no more than 25% of the bone marrow was irradiated. Radiotherapy should have been completed at least 4 weeks before inclusion, and patients should have recovered from any toxic side effect. The irradiated area was excluded from tumour measurements. All patients had to have a performance status ⩽2 according to the Eastern Cooperative Oncology Group (ECOG) scale and a life expectancy of at least 12 weeks. An adequate bone marrow reserve (leucocytes ⩾3.0 × 10^9^ l^−1^, neutrophils ⩾1.5 × 10^9^ l^−1^, platelets ⩾100 × 10^9^ l^−1^, haemoglobin ⩾6.2 mmol l^−1^), normal renal (serum creatinine ⩽120 *μ*mol l^−1^ or creatinine clearance ⩾60 ml min^−1^) and liver function (serum bilirubin ⩽35 *μ*mol l^−1^, serum alanine aminotransferase (ALAT), and serum aspartate aminotransferase (ASAT) less than three times the upper limit of normal) were required. Patients were excluded if they had active infections, second primary malignancies (except carcinoma *in situ* of the cervix, adequately treated basal cell carcinoma of the skin, and adequately treated upper respiratory tract malignancy), uncorrected hypercalcaemia, or an LVEF ⩽45% measured by multiple gated acquisition (MUGA) scan. Local medical ethics committees of all hospitals approved the protocol. All patients gave informed consent before study entry.

### Treatment

Eligible patients were randomised by telephone to receive either cisplatin or epirubicin both with gemcitabine. Gemcitabine (1125 mg m^−2^) was administered in 250 ml 0.9% NaCl by a 30 min infusion on days 1 (before cisplatin or epirubicin) and 8. Cisplatin 80 mg m^−2^ (in 1000 ml 0.9% NaCl) was administered intravenously during 3 h after prehydration with 0.9% NaCl on day 2 of each 21-day treatment cycle. Epirubicin 100 mg m^−2^ (in 50 ml 0.9% NaCl) was administered as an intravenous bolus injection within 5 min on day 1 of each 21-day cycle. For prehydration, patients in the CG arm were admitted to hospital for 2 days. Epirubicin–gemcitabine was administered as an outpatient regimen. Anti-emetics consisted of ondansetron 8 mg and dexamethasone 8 mg twice a day on days 1, 2, and 8. The treatment consisted of a maximum of five cycles and was stopped earlier in case of tumour progression, intolerable toxicity, or patient's wish. No treatment with G-CSF was foreseen. In case of Hb<5.0 mmol l^−1^ or symptomatic anaemia in combination with Hb<6.0 mmol l, patients were treated with red blood cell transfusion. Platelet transfusion was given in the event of platelets <10 × 10^9^ l^−1^ or persistent bleeding in combination with platelets <20 × 10^9^ l^−1^.

### Dose adjustments

Drug administration was postponed to a maximum of 2 weeks if there was no haematologic recovery on day 22 (neutrophils <1.5 × 10^9^ l^−1^ and/or platelets <100 × 10^9^ l^−1^) or in case of persistent common toxicity criteria (CTC) grade 2 or more nonhaematologic toxicity (except alopecia). The dose of cisplatin or epirubicin for subsequent cycles was reduced to 75% in case of a nadir of neutrophils below 0.5 × 10^9^ l^−1^ exceeding 7 days, a nadir of platelets below 25 × 10^9^ l^−1^, thrombocytopenia associated with bleeding, febrile neutropenia, or CTC grade 3 nonhaematologic toxicity (except nausea and vomiting). The cisplatin dose was reduced by 50% for a calculated creatinine clearance between 50 and 70 ml min^−1^, and in case of a creatinine clearance less than 50 ml min^−1^ cisplatin was not administered (Cockcroft and Gault, 1976). The dose of gemcitabine on day 8 was reduced to 50% in case of neutrophils between 0.5 and 1.5 × 10^9^ l^−1^, platelets between 50 and 100 × 10^9^ l^−1^ or grade 3 nonhaematologic toxicity on day 8. Gemcitabine was omitted on day 8 in case of neutrophils <0.5 × 10^9^ l^−1^, platelets <50 × 10^9^ l^−1^ or grade 4 nonhaematologic toxicity. The mean relative dose intensity was calculated by dividing the delivered dose (mg m^−2^ week^−1^) by the planned dose (mg m^−2^ week^−1^) for the number of cycles each patient received.

### Treatment evaluation

Complete blood cell counts were performed at least on days 1 and 8 of each cycle. On day 1 of each cycle, patient evaluation also included liver and renal functions, performance status, and toxicity scoring according to the CTC of the National Cancer Institute. The LVEF was measured by the MUGA scan before and 6–12 weeks after treatment. All patients were evaluable for toxicity. The tumour response was evaluated by the treating physician, at least after three and five cycles, according to the World Health Organisation (WHO) criteria (WHO, 1979). After treatment, tumour responses were evaluated by an independent observer.

After discontinuation of treatment, patients were evaluated every 6 weeks with physical examination, laboratory tests, chest X-ray or CT-scan of the chest, and additional imaging tests on clinical indication to assess tumour progression.

At the start of treatment, after three cycles of chemotherapy, and 6 weeks after the end of treatment, QOL was measured with the European Organisation for Research and Treatment of Cancer (EORTC) QLQ-C30, supplemented by a 13-item lung-cancer-specific questionnaire module, the EORTC QLQ-LC13. This validated questionnaire was filled in at home and is composed of a core QOL questionnaire covering general aspects of health-related QOL and disease- and treatment-specific symptoms (Aaronson *et al*, 1993; Bergman *et al*, 1994).

### Statistical analysis

The primary end point of the study was progression-free survival. The study was designed to detect a 20% increase in 6-month progression-free survival, from 40% in the CG arm to 60% in the EG arm. To detect such an increase using a two-sided 0.05 alpha-level test with 85% power, the required accrual was determined to be 120 patients in both arms. Analysis was performed on the intention-to-treat principle. The time from the date of randomisation to the date of first documented progression was defined as progression-free survival. Overall survival was defined as the interval between the date of randomisation to the date of death. Progression-free and overall survivals for both treatment arms were compared by Kaplan–Meier curves using the log-rank test. Quality of life was analysed by ANOVA for the different functional areas and symptoms at all three points of measurement. To identify potential prognostic factors, a multivariate analysis was performed using a logistic regression model for response rate and a Cox regression model for progression-free and overall survivals. A *P*<0.05 was considered statistically significant.

## RESULTS

### Patient characteristics

Between November 1998 and February 2002, 240 patients from four hospitals in the Northern part of the Netherlands were recruited. Patients were randomised to CG (*n*=119) or EG (*n*=121). Patient characteristics were not significantly different between both treatment arms (Table 1

**Table 1:** Patient characteristics

	**Cisplatin–gemcitabine**		**Epirubicin–gemcitabine**		
	**No.**	**%**	**No.**	**%**	***P***
Patients entered	119	50	121	50	ns
					
*Sex*					ns
Male	94	79	85	70	
Female	25	21	36	30	
					
*Age, years*					
Median	60		60		
Range	29–80		32–76		ns
					
*Stage*					ns
IIIa	8	7	8	7	
IIIb	43	36	44	36	
IV	68	57	69	57	
					
*Performance status*					ns
0	36	30	37	31	
1	66	55	73	60	
2	17	14	11	9	
					
*Histology*					ns
Squamous cell carinoma	41	35	32	26	
Adenocarcinoma	41	35	50	41	
Large cell carcinoma	36	30	38	31	
Other	1	1	1	1	
					
*Weight loss (last 3 months)*					ns
<5%	82	69	80	66	
⩾5%	37	31	41	34	
					
*Liver metastases*					ns
Absent	109	92	111	92	
Present	10	8	10	8	

ns=Not significant.

). Seven patients had been treated with prior radiotherapy on the primary tumour. Three randomised patients did not receive chemotherapy because of rapidly deteriorating performance status due to progression of disease before treatment initiation. These patients were included in all analyses.

### Toxicity

Haematologic toxicity is shown in Table 2

**Table 2:** Worst haematologic CTC toxicity grade per patient

	**Cisplatin–gemcitabine**		**Epirubicin–gemcitabine**		
**Toxicity**	**No.**	**%**	**No.**	**%**	***P***
*Anaemia*					ns
1	20	17	18	16	
2	77	66	68	60	
3/4	20	17	26	23	
					
*Leukopenia*					<0.01
1	24	21	9	8	
2	48	41	31	27	
3/4	27	23	68	60	
					
*Granulocytopenia*					<0.01
1	18	19	10	12	
2	22	23	8	10	
3/4	31	32	55	65	
					
*Thrombocytopenia*					ns
1	19	16	35	31	
2	22	19	24	21	
3/4	66	56	48	42	
					
*Transfusions*					
Red blood cells^a^	66	56	61	50	ns
Platelets^a^	9	8	11	9	ns
*Febrile neutropenia*	2	2	13	11	<0.01

a Patients receiving at least one red blood cell or platelet transfusion.

ns=Not significant.

. In the EG arm, grade 3 or 4 leukopenia and granulocytopenia occurred more frequently as compared to the CG arm. Epirubicin–gemcitabine patients were more often hospitalised for febrile neutropenia than patients in the CG arm (11 *vs* 2% of patients, *P*=0.006). In both arms, two patients died due to septicaemia (EG arm after one and three cycles, CG arm after three and five cycles). In the CG arm, 56% of patients had one or more red blood cell transfusions during treatment, compared to 51% of patients in the EG arm (*P*=0.433). At the time of study, erythropoietin was not routinely administered. The number of red blood cell transfusions per patient was also not different between both treatment arms.

The worst nonhaematologic toxicity per patient is listed in Table 3

**Table 3:** Worst nonhaematologic CTC toxicity grade per patient

	**Cisplatin–gemcitabine**		**Epirubicin–gemcitabine**		
**Toxicity**	**No.**	**%**	**No.**	**%**	***P***
*Bilirubin*					ns
1	5	4	10	10	
2	0	0	1	1	
3/4	0	0	2	2	
					
*ASAT*					<0.01
1	31	27	43	40	
2	0	0	8	7	
3/4	0	0	4	4	
					
*ALAT*					<0.01
1	50	44	46	43	
2	12	10	27	25	
3/4	2	2	14	13	
					
*Creatinine*					<0.01
1	53	45	10	9	
2	9	8	4	4	
3/4	0	0	0	0	
					
*Mucositis*					<0.01
1	15	13	23	20	
2	7	6	46	40	
3/4	0	0	14	12	
					
*Nausea*					<0.01
1	36	31	46	40	
2	51	44	27	24	
3/4	5	4	1	1	
					
*Vomiting*					<0.01
1	37	32	28	25	
2	27	23	11	10	
3/4	2	2	1	1	
					
*Diarrhoea*					ns
1	6	5	15	13	
2	4	3	2	2	
3/4	3	3	1	1	
					
*Fever*					<0.01
1	11	9	22	19	
2	2	2	12	10	
3/4	1	1	3	3	
					
*Infections*					ns
1	0	0	1	1	
2	2	2	3	3	
3/4	9	8	13	11	
					
*Skin reactions*					ns
1	9	8	14	12	
2	7	6	1	1	
3/4	1	1	2	2	
					
*Sensory neuropathy*					<0.01
1	16	14	2	2	
2	7	6	1	1	
3/4	0	0	0	0	
					
*Motoric neuropathy*					ns
1	0	0	0	0	
2	2	2	2	2	
3/4	1	1	1	1	
					
*Anorexia*					ns
1	50	43	46	40	
2	27	23	38	33	
3/4	2	2	0	0	
					
*Fatigue*					<0.05
1	41	35	22	19	
2	54	46	57	50	
3/4	5	4	5	4	

ns=Not significant.

. Gemcitabine can induce short-lasting elevation of transaminases, this was experienced more frequently in the EG arm (*P*=0.001). Patients in the EG arm also had more often short-lasting fever (in the absence of neutropenia). Nausea, vomiting, and fatigue frequently occurred in both arms, but more often in the CG arm. Significantly more patients in the EG arm had a grade 1 (16% in the CG arm *vs* 42% in the EG arm) and grade 2 (7% in the CG arm *vs* 13% in the EG arm) decline in LVEF (*P*=0.006, *n*=69). Clinically evident cardiac failure was not observed during follow-up. In this trial, 28 elderly patients (⩾70 years) were included. In these patients, grade 3 or 4 thrombocytopenia occurred more frequently compared to younger patients, in 78 *vs* 46% of patients, respectively (*P*=0.029). No differences in other toxicities were found when comparing them to younger patients.

### Treatment

The median (range) number of cycles per patient was 4 (0–5) for both arms. The maximum of five cycles was completed in 58 (49%) patients in the CG arm and in 49 (41%) patients in the EG arm (Table 4

**Table 4:** Number of chemotherapy cycles per patient

	**Cisplatin–gemcitabine**		**Epirubicin–gemcitabine**	
**No. of cycles**	**No.**	**%**	**No.**	**%**
0	1	1	2	2
1	11	9	19	16
2	8	7	11	9
3	18	15	20	17
4	23	19	20	17
5	58	49	49	41
Median (range)	4 (0–5)		4 (0–5)	

). The reasons for treatment discontinuation between both arms were not different (Table 5

**Table 5:** Reasons for chemotherapy discontinuation

	**Cisplatin–gemcitabine**		**Epirubicin –gemcitabine**	
**Reason for discontinuation**	**No.**	**%**	**No.**	**%**
Progressive disease	18	15	24	20
Toxicity	21	18	27	22
Death due to toxicity	1	1	2	2
Unrelated death	2	2	3	3
Patient's request	7	6	5	4
Other	12	10	11	9
Total discontinuations	61	51	72	60

).

The mean relative dose intensity for cisplatin and epirubicin was 94 and 95%, respectively. The mean relative dose intensity of gemcitabine was not significantly different between both treatment arms; 89% for the EG arm and 92% for the CG arm. Cisplatin, epirubicin, and gemcitabine doses were reduced in, respectively, 6, 5, and 12% of cycles (for gemcitabine, days 1 and 8 were taken together).

### Tumour response

The overall response rate was 46% (95% CI, 37–55) for the CG arm and 36% (95% CI, 28–45) for the EG arm (*P*=0.121) (Table 6

**Table 6:** Tumour response

	**Cisplatin–gemcitabine**		**Epirubicin–gemcitabine**		
**Response**	**No.**	**%**	**No.**	**%**	***P***
Complete response	1	1	0	0	
Partial response	54	45	44	36	
Stable disease	40	34	37	31	
Progressive disease	22	19	26	22	
Not assessable	2	2	14	12	
					
Overall response (95% CI)		46 (37–55)		36 (28–45)	0.121

). In all, 13 patients were not evaluable for tumour response due to early death from toxicity (*n*=1), death from other causes (*n*=4), discontinuation of treatment at patient's request after one cycle (*n*=4), toxicity (*n*=2), and for other reasons (*n*=2). Three patients did not receive chemotherapy at all and were, therefore, not evaluable for response. For the analysis, these patients were considered as nonresponders. The response rates for patients with stage III *vs* stage IV disease were 55% (95% CI, 41–69) and 40% (95% CI, 28–51) in the CG arm, and 44% (95% CI, 31–58) and 30% (95% CI, 20–41) in the EG arm, respectively. All these differences were not statistically significant. The tumour response rate in patients with a performance status of 0 or 1 was 43% compared to 36% in patients with a performance status of 2 (*P*=0.443). Elderly patients (⩾70 years) had a similar tumour response rate compared to younger patients. However, patients with liver metastases (*n*=20) had a lower response rate (15%) compared to patients without these metastases (44%) (*P*=0.013). A logistic regression model with potential prognostic factors (disease stage, histology, sex, age, performance status, weight loss, and liver metastases) showed that the presence of liver metastases was a significant independent negative prognostic factor for tumour response.

A total of 38 patients in the CG arm (32%) and 39 patients in the EG arm (32%) received second-line chemotherapy, consisting of docetaxel alone or docetaxel combined with carboplatin or irinotecan. A partial response to second-line therapy was observed in 20% of patients, without a difference between CG and EG. In all, 24% of responders and 14% of nonresponders on first-line therapy achieved a partial response to second-line therapy; this difference was also not significant. Median survival after start of second-line chemotherapy (*n*=77) was 28 weeks (95% CI, 22–34).

### Progression-free survival and overall survivals

**Figure 1:**
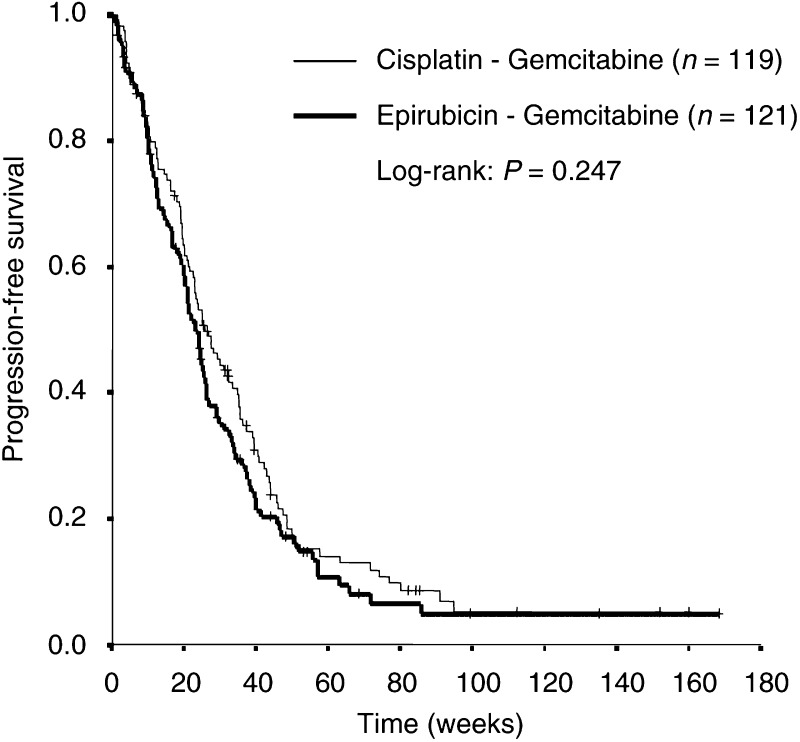
Kaplan–Meier curve for progression-free survival

**Figure 2:**
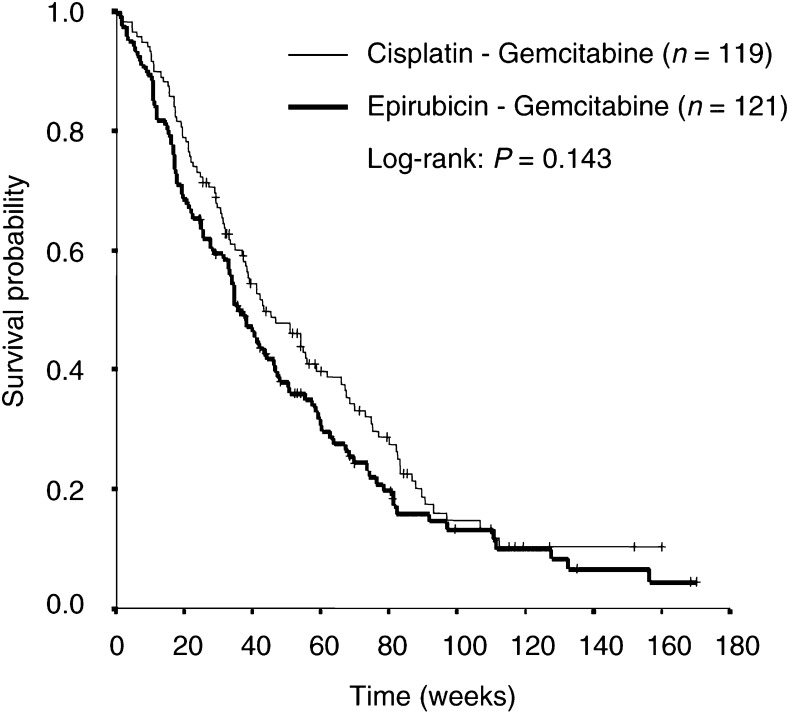
Kaplan–Meier curve for overall survival

On August 2002, 47 patients were still alive. The median progression-free survival was not significantly different between both treatment arms; 26 (95% CI, 20–31) *vs* 23 (95% CI, 20–26) weeks for the CG and EG arm, respectively (*P*=0.247). Progression-free survival (±s.e.) at 6 months follow-up was 49% (±5%) *vs* 40% (±5%) for the CG and EG arm, respectively (*P*=0.134) (Figure 1

). The median overall survival was 43 (95% CI, 30–57) weeks in the CG arm, *vs* 36 (95% CI, 30–42) weeks in the EG arm, which was not different between both arms (*P*=0.143). The 1-year survival rate (±s.e.) was 45% (±5%) *vs* 35% (±5%) in the CG and EG arm, respectively (*P*=0.123) (Figure 2

). Progression-free and overall survivals were not different in elderly patients (⩾70 years) when compared to younger patients.

A Cox regression model was used to find independent prognostic factors for progression-free and overall survivals. The factors disease stage, histology, sex, age, performance status, weight loss, and liver metastases were used in this model. Performance status ⩽1, absence of liver metastases, and weight loss <5% were associated with prolonged progression-free and improved overall survivals. Stage III disease was, compared to stage IV disease, associated with longer progression-free survival.

### Quality of Life

The questionnaires filled in at the patient's home at the start of treatment, after three cycles of chemotherapy, and 6 weeks after the end of treatment were returned to the datacenter by 70, 47, and 45% of patients, respectively. Between both treatment arms, no significant differences were found in global health status and functional scales (physical, role, emotional, cognitive, and social functioning), at all three moments of measurement (Table 7a

**Table 7:** Mean quality of life scores before, during, and after treatment

	**Cisplatin–gemcitabine**		**Epirubicin–gemcitabine**		
**Items**	**Mean**	**s.e.**	**Mean**	**s.e.**	***P***
(a)					
*Global health status*^a^					
Before	53.1	2.7	58.1	2.4	ns
During	53.4	2.7	53.5	3.0	ns
After	52.0	2.9	51.7	3.1	ns
					
**Functional scales^a^**					
*Physical*					
Before	59.1	3.5	67.3	2.9	ns
During	56.3	3.6	51.2	3.8	ns
After	57.0	3.9	47.1	3.6	ns
					
*Role*					
Before	53.2	3.7	59.3	3.3	ns
During	48.0	3.8	37.7	4.2	ns
After	45.9	3.7	43.0	4.1	ns
					
*Cognitive*					
Before	82.1	2.5	82.7	2.6	ns
During	80.4	3.0	77.7	3.6	ns
After	79.9	3.0	81.2	2.8	ns
					
*Emotional*					
Before	62.4	2.6	64.0	2.6	ns
During	72.4	3.3	69.6	3.3	ns
After	72.5	2.6	72.9	2.8	ns
					
*Social*					
Before	73.6	2.8	74.0	3.0	ns
During	64.9	3.9	65.7	4.0	ns
After	67.3	3.8	65.2	4.1	ns
					
(b)					
**Symptom scales^b^**					
*Fatigue*					
Before	40.2	3.3	37.3	3.0	ns
During	49.2	3.2	56.4	3.7	ns
After	46.5	3.5	52.0	3.8	ns
					
*Nausea and vomiting*					
Before	11.0	2.1	7.3	1.4	ns
During	32.5	4.3	16.7	2.9	0.003
After	31.5	4.5	12.7	3.5	0.001
					
*Pain*					
Before	25.4	3.1	27.8	3.1	ns
During	16.7	3.3	26.2	4.3	ns
After	22.2	3.9	32.4	4.4	ns
					
*Appetite loss*					
Before	24.4	3.5	21.6	3.2	ns
During	36.2	4.6	37.0	4.6	ns
After	30.8	4.3	30.2	4.6	ns
					
*Sore mouth*					
Before	8.2	2.3	9.4	2.5	ns
During	16.7	3.4	42.3	5.3	0.001
After	9.4	3.2	35.2	5.6	0.001
					
*Dysphagia*					
Before	8.5	2.1	11.0	2.5	ns
During	13.6	3.4	31.4	5.0	0.003
After	9.4	2.9	27.2	4.8	0.002
					
*Peripheral neuropathy*					
Before	11.0	2.4	7.1	1.9	ns
During	16.4	3.3	14.0	3.2	ns
After	19.5	4.0	12.6	3.0	ns

a Scores range from 0 to 100, with a higher score representing a higher level of functioning.

b Scores range from 0 to 100, with a higher score representing a greater degree of symptoms.

ns=Not significant.

). On symptom scales, differences were found in the occurrences of nausea and vomiting, that were more common in the CG arm. A sore mouth and dysphagia were more frequently noted in the EG arm. Fatigue did not score different between both treatment arms. A selection of scores on symptom scales from the EORTC QLQ-C30 and QLQ-LC13 is shown in Table 7b.

## DISCUSSION

The efficacy of epirubicin and cisplatin both combined with gemcitabine is not significantly different in terms of progression-free survival and tumour response rates. Since overall survival was not the primary end point of this trial and our power analysis was not performed to detect differences in overall survival, no definitive conclusions in terms of overall survival can be drawn although we found no significant differences in overall survival between both arms. The overall toxicity was different and more severe in the epirubicin combination. Neutropenia, febrile neutropenia, elevation of serum transaminases, mucositis, and fever occurred more frequently in the EG arm while nausea, vomiting, nephrotoxicity, and sensory neuropathy were more common in the CG arm. The decline in LVEF after treatment, especially grade 1, was more evident in the EG arm, but was not associated with clinical signs of heart failure during follow-up. In line with CTC toxicity evaluation, the symptom scales of EORTC questionnaires showed a different spectrum of toxicity in both arms. The global health status and scores on functional scales were similar in both arms in all three moments of measurement, although the number of patients who returned their questionnaires was limited and therefore no firm conclusions can be drawn.

Although in this trial efficacy was similar in both arms in terms of progression-free survival, overall survival, and response rate, this trial failed to show benefits in terms of less toxicity for the nonplatinum-based regimen. Other trials showed similar response rates and survival, and less toxicity in patients treated with nonplatinum-based schedules compared to platinum-containing combinations (Gatzemeier *et al*, 1991; Gridelli *et al*, 1996; Georgoulias *et al*, 2001; Sculier *et al*, 2002). A recently published study compared four platinum-based regimens for advanced NSCLC (cisplatin with either paclitaxel, gemcitabine, or docetaxel, and carboplatin with paclitaxel), and showed no difference in survival (the median survival was 7.9 months for all patients) and response rate (19% for all patients). However, progression-free survival was longer in the group of patients who received cisplatin and gemcitabine (4.2 months). On the other hand, treatment with cisplatin and gemcitabine was associated with more renal toxicity (Schiller *et al*, 2002). Recently, Rosell *et al* (2002) compared two platinum-based regimens and showed that a cisplatin-based therapy was associated with a significantly longer median survival. However, in our trial, response rates and survival were similar in both arms, but the nonplatinum-containing regimen had a less favourable toxicity profile. Based on these data, we conclude that a platinum-based combination therapy remains the standard treatment for advanced NSCLC.

A performance status of 2, liver metastases, and weight loss of more than 5% were associated with a worse survival. An unfavourable survival outcome for patients with a poor performance status has also been reported in other studies (Comella *et al*, 1996; Cullen *et al*, 1999; Le Chevalier *et al*, 2001; Sweeney *et al*, 2001). Whether chemotherapy in patients with a performance status of 2 should be advocated is still a matter of debate. For these patients, a new therapeutic approach, for example, with biologicals with hardly any toxicity, may be a more attractive treatment option. Further trials are required to investigate these modalities.

In the 28 elderly patients (⩾70 years) included in this trial toxicity (except thrombocytopenia), the tumour response rate, progression-free and overall survivals were not different compared to younger patients. However, in contrast to the fact that 35–43% of all lung cancers arise beyond the age of 70 years (Fry *et al*, 1999), in this trial only 12% of patients were over 70 years of age. It is possible that we cannot demonstrate differences between elderly and younger patients due to exclusion of elderly patients with a poor performance status or comorbidity. Generally, elderly patients are thought to be less able to tolerate polychemotherapy (Frasci, 2002). However, to date prospective randomised trials on the beneficial role of platinum-based chemotherapy in elderly NSCLC patients are not available (Frasci, 2002).

Shepherd *et al* (2000) have shown that second-line treatment with single-agent docetaxel, compared to best supportive care, is associated with prolongation of survival. The tumour response rate of single-agent docetaxel in this trial was 7.1%. However, in our study the observed tumour response rate after predominantly docetaxel-containing doublets as second-line chemotherapy regimens was 20%, suggesting that a combination regimen may be more effective than a single agent one. Other trials on second-line chemotherapy in NSCLC, reviewed by Huisman *et al* (2000), also report higher response rates.

In conclusion, we found no differences in efficacy and global QOL between cisplatin and epirubicin, both in combination with gemcitabine as first-line treatment for advanced NSCLC. However, the observed differences in toxicity profile are in favour of cisplatin. Therefore, a platinum-based combination regimen remains the recommended treatment for advanced NSCLC.
